# Prevalence of Functional Gastrointestinal Disorders according to Rome III Criteria in Italian Morbidly Obese Patients

**DOI:** 10.1155/2013/532503

**Published:** 2013-11-04

**Authors:** Antonella Santonicola, Luigi Angrisani, Carolina Ciacci, Paola Iovino

**Affiliations:** ^1^Department of Clinical Medicine and Surgery, University Federico II of Naples, 80131 Naples, Italy; ^2^General and Endoscopic Surgery Unit, S. Giovanni Bosco Hospital, 80144 Naples, Italy; ^3^Gastrointestinal Unit, Department of Medicine and Surgery, University of Salerno, Baronissi, 84081 Salerno, Italy

## Abstract

The relationship between GI symptoms and obesity has yet to be completely clarified. *Aim*. To determine in a morbidly obese southern Italy adult population the prevalence of Functional Gastrointestinal Disorders (FGID) and its association with the presence of a Binge Eating (BE) behavior pattern. *Methods*. Consecutive obese patients eligible for bariatric surgery and 100 Healthy Controls (HC) were recruited. All participants were questioned and scored for the presence of FGID according to Rome III criteria and for the presence or the frequency-intensity of a number of upper and lower GI symptoms. BE behavior pattern was assessed. *Results*. One-hundred obese patients met the inclusion criteria. The prevalence of FGID was similar between obese patients and HC. There was a significant association between obese patients with BE behavior and postprandial distress syndrome (*P* = 0.04). Moreover, a significantly higher frequency-intensity score for epigastric fullness (1.23 ± 0.45 versus 0.35 ± 0.13, *P* = 0.01) was found in obese patients with BE behavior compared to obese patients without. *Conclusions*. Obese patients with a BE behavior pattern showed a significantly higher prevalence of postprandial distress syndrome. A greater knowledge of the GI symptoms associated with obesity along with the pathophysiological mechanisms underlying will be important in the clinical management of these patients.

## 1. Introduction

Obesity is considered a multifactorial disease, that results from a mixture of genetic predisposition, environmental influences (e.g., sedentary lifestyle), and behavioral components (e.g., food as a reward) [1]. Being overweight and obese are well-known causes of morbidity and mortality [2–5]. In the United States, the obesity epidemic is one of the major issues with significant health, social, and economic implications [6]. The rate of obesity has more than doubled over the past 30 years, and also in most countries of northern Europe including the UK and Scandinavian countries, as well as selected southern European countries [7]. Globally, the prevalence of obesity in the European adult population is about 15.5% [8]. Actually, among European countries, Italy has the lowest adult obesity prevalence [7–9], at about 9% (8.5% men, 9.4% women) [7]. However, within Italy, differences in BMI have been reported according to geographic area; in fact, the prevalence of obesity is approximately two-fold higher in southern compared to northern Italy [10]. The underlying causes of these differences are under evaluation, but different socioeconomic status, lifestyle, and dietary habits may play a role [7].

Although the Gastrointestinal (GI) tract is the dominant organ system associated with food intake, the relationship between GI symptoms and obesity has yet to be completely clarified. Previous studies described a greater prevalence of symptoms fulfilling criteria for irritable bowel syndrome (IBS) and gastroesophageal reflux disease (GERD) in morbidly obese patients compared to the general population [11]. Other authors showed a positive relationship between BMI and frequent vomiting, upper abdominal pain, bloating, and diarrhea [12]. Fysekidis et al. reported a higher prevalence of functional upper and lower disorders in their obese population [13]. A recent meta-analysis reported no significant association between constipation/hard stools or bloating and increasing BMI [14].

Among severely obese patients undergoing bariatric surgery, a large prevalence of binge eating (BE) disorders was found [15]. BE disorders have already been associated with the experience of both upper and lower GI symptoms [15, 16]. 

To our knowledge, there is a lack of studies investigating both GI symptoms and BE disorders in an Italian obese population. 

Therefore, the current study aimed to the following:

- determine in a morbidly obese southern Italy adult population the prevalence of Functional Gastrointestinal Disorders (FGID) such as Functional Dyspepsia (FD), Irritable Bowel Syndrome (IBS), Functional Constipation, Functional Diarrhea, and Functional Bloating according to the international accepted Rome III criteria [17] and the frequency-intensity scores of a broader number of GI symptoms in this population,
- evaluate the association between FGID and the presence of BE behavior.

## 2. Methods 

### 2.1. Population

One hundred and twenty-eight consecutive obese patients were recruited from an outpatient clinic devoted to the surgical therapy of obesity and related disorders at the San Giovanni Bosco Hospital, Naples, Italy. Obese patients were eligible for bariatric surgery fulfilling the Italian consensus inclusion criteria for bariatric surgery [18]: body mass index (BMI) more than 35 kg/m^2^ with at least one known comorbidity (hypertension, diabetes, sleep apnea) or a BMI >40 kg/m^2^. The study was approved by the Institutional Review Board of San Giovanni Bosco Hospital, Naples, Italy. Adherence to the ethical conduct standards of the Declaration of Helsinki ensured patients' welfare [19]. Informed consent was obtained from all patients.

Patients' enrolment lasted from June 2012 to April 2013 and involved all obese patients meeting the inclusion/exclusion criteria. Inclusion criteria were as follows: Caucasian adults from the South of Italy; age from 18 to 60 years; no previous gastrointestinal surgery, with the exception of appendectomy. Exclusion criteria were as follows: the use of oral contraceptives, oral corticosteroid treatment, pregnancy, major psychiatric disorders, drug and alcohol abuse, and any other organic gastrointestinal diseases.

Demographic characteristics (gender, age, smoking habits, school degree), anthropometric data (weight, height and BMI), and prevalence of comorbidities, that is, hypertension, dyslipidemia, type II diabetes mellitus [20], and respiratory diseases were collected for each of the obese patients.

One hundred healthy controls (HC) were enrolled from friends of obese patients and hospital staff and used as controls as a comparison to obese patients. Inclusion criteria for HC were Caucasian adults from the southern Italian region Campania, aged between 18 to 60 years. Exclusion criteria were similar to that for obese patients. Demographic characteristics (gender, age, smoking habits, school degree) and anthropometric data (weight, height and BMI) were collected for each HC.

### 2.2. Questionnaires

#### 2.2.1. Functional Gastrointestinal Disorders Questionnaires

All participants were questioned and scored both on the validated questionnaires testing the presence of FGID according to Rome III criteria [17] together with the exclusion of any organic disease and on previously published questionnaires dealing with the presence or the frequency-intensity score of a number of upper and lower GI symptoms.

- The FD questionnaire consisted of 18 questions and allowed the diagnosis of FD and its two subgroups: the Postprandial Distress Syndrome (PDS) and the Epigastric Pain Syndrome (EPS). The characteristic symptoms of PDS were bothersome postprandial fullness or early satiation and those of EPS were unexplained epigastric pain or burning [17, 21].
- Frequency-intensity scores were calculated for the 4 cardinal symptoms pragmatically described by the Rome III Committee such as early satiation, epigastric fullness, epigastric pain, and burning together with other upper GI symptoms such as epigastric pressure, belching, nausea, vomiting, regurgitation, heartburn, noncardiac chest pain, and cough, with a scoring frequency from 0 to 3 (0 = absent, 1 = 2 d/wk; 2 = 3–5 d/wk; and 3 = 6 or 7 d/wk) and intensity from 0 to 3 (0 = absent; 1 = not very bothersome, not interfering with daily activities; 2 = bothersome, but not interfering with daily activities; and 3 = interfering with daily activities). A frequency-intensity score from 0 up to a maximum of 6 was obtained for each symptom [22, 23].
- For IBS, Functional Constipation, Functional Diarrhea, and Functional Bloating, questionnaires were used. IBS and its subtypes: IBS predominant constipation (IBS-C), IBS predominant (IBS-D), mixed IBS (IBS-M), and unclassified IBS (IBS-U) were further evaluated.
- Frequency-intensity score for bloating was calculated as described above [22, 23].
- Other abdomino-pelvic symptoms such as leakage of stool, the sensations of incomplete evacuation, anal blockage, flatulence, urgency, and straining were evaluated by binomial answer (yes/no) [24].
- Daily measurement of the number of bowel movements was summarized weekly.

#### 2.2.2. Binge Eating Behavior Pattern Questionnaires

A psychologist performed a structured interview for psychological assessment and administered the questionnaires. BE behavior pattern was assessed according to an expanded Structured Clinical Interview for the Diagnostic and Statistical Manual, Fourth Edition (SCID) [25], using the following two questions.

“Have you ever had eating binges when you ate what most people would regard as an unusually large amount of food in a short period of time?” (“yes,” “no,” and “do not know/refuse”) and “when you were having eating binges, did you feel that your eating was out of control?” (“not at all,” “slightly,” “somewhat,” “very much,” “extremely,” and “do not know/refuse”). In the current study, the BE pattern was scored as present if respondents answered “yes” to the first question and any loss of control to the second question. If respondents answered “no” to the first question or “not at all” to the second question, the BE pattern was scored as absent.

#### 2.2.3. Statistical Analysis

Percent frequencies and means with respective standard errors (Mean ± SE) were calculated for sample descriptive statistics, unless otherwise specified. Unpaired Student's *t*-test was used to compare continuous data and *χ* ^2^ for categorical data. Pearson's correlation (*r*) was used as appropriate. Statistical significance was accepted as *P* < 0.05. The statistical program used was the Statistical Package for the Social Sciences (SPSS) for Windows, version 15.0. 

## 3. Results

Twenty-eight obese patients were excluded on the basis of one or more exclusion criteria: 1 patient for the concomitant treatment for oral contraceptives, 1 patient for the concomitant oral corticosteroid treatment for Systemic Lupus Erythematosus, 10 patients for previous cholecystectomy, and 1 patient for a diagnosis ulcerative colitis. Upper GI endoscopy revealed one peptic ulcer and 14 erosive esophagitis, so 15 further patients were consequently excluded.

One hundred obese patients were finally enrolled.

Demographic characteristics, anthropometric data, and prevalence of comorbidities of obese patients and HC are shown in Table 1.

Eleven/100 (11%) obese patients and 11/100 HC (11%) fulfilled the diagnosis of FD according to Rome III criteria (*P* = 1.0). In detail, 4 obese patients and no HC fulfilled the diagnostic criteria for EPS, whilst 7 obese patients and 11 HC did for PDS (*P* = 0.5).

**Figure 1:**
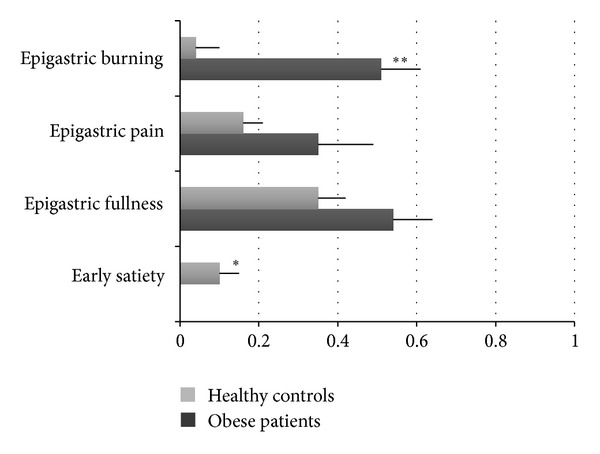
This figure shows the frequency-intensity scores (M ± SE) of the 4 cardinal symptoms: early satiation, epigastric fullness, epigastric pain, and epigastric burning in the studied obese population and HC.

Frequency-intensity scores for the 4 cardinal symptoms such as early satiation, epigastric fullness, epigastric pain, and epigastric burning in the obese population and HC are reported in Figure 1.

**Figure 2:**
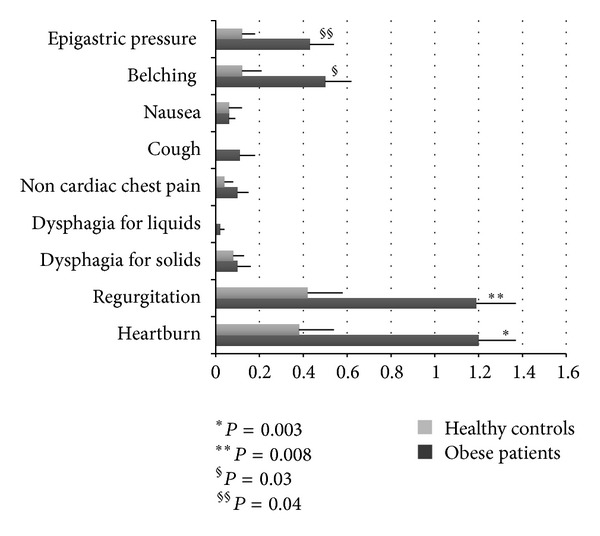
This figure shows the frequency-intensity scores (M ± SE) of GI symptoms: epigastric pressure, belching, nausea, cough, noncardiac chest pain, dysphagia for liquids, dysphagia for solids, regurgitation, and heartburn in obese patients and HC.

The frequency intensity scores of the other upper GI symptoms such as epigastric pressure, belching, nausea, cough, non-cardiac chest pain, dysphagia for liquids, dysphagia for solids, regurgitation, and heartburn in obese patients and HC are illustrated in Figure 2.

A significant increase in typical GERD symptoms, such as heartburn and regurgitation, in epigastric pressure and belching intensity frequency scores was found in obese patients compared to HC. 

There was a significant correlation between the frequency-intensity scores of heartburn and regurgitation and epigastric burning (*r* = 0.57, *P* < 0.001 and *r* = 0.51, *P* < 0.001, resp.) and bloating (*r* = 0.31, *P* = 0.002 and *r* = 0.23, *P* = 0.02, resp.).

FD was found in 4/22 (18.2%) of obese patients with BE behavior and 7/71 (9.0%) of obese patients without BE behavior (*P* = 0.22) according to Rome III criteria. There was a significant association between obese patients with BE behavior and PDS, while no obese patients with BE behavior fulfilled the diagnostic criteria for EPS (*P* = 0.04). Obese patients with BE behavior showed significantly higher frequency-intensity scores for epigastric fullness (1.23 ± 0.45 versus 0.35 ± 0.13, *P* = 0.01) compared to obese patients without BE behavior.

Table 2 describes the frequency intensity scores of the studied upper GI symptoms in obese patients with and without BE behavior pattern. Among them, only nausea frequency-intensity score was borderline significantly higher in obese with BE than in obese without BE.

Stool frequency weekly summarized was 13.6 ± 0.8 in obese patients and 6.6 ± 0.3 in HC (*P* < 0.001). IBS according to Rome III criteria was diagnosed in 12/100 (11%) of obese patients and 22/100 (22%) of HC (*P* = 0.09). In detail, 8 obese patients and 10 HC fulfilled the diagnostic criteria for IBS-C; 3 obese patients and 10 HC for IBS-D and 1 obese patient and 2 HC for IBS-M (*P* = 0.46). 

Functional Constipation was found in 8/100 (8%) obese patients and 6/100 (6%) HC diagnosed on the basis of the Rome III criteria; one obese patient and no HC fulfilled the diagnostic criteria for Functional Diarrhea and 2/100 (2%) obese patients and no HC for Functional Bloating (*P* = 0.17).

**Figure 3:**
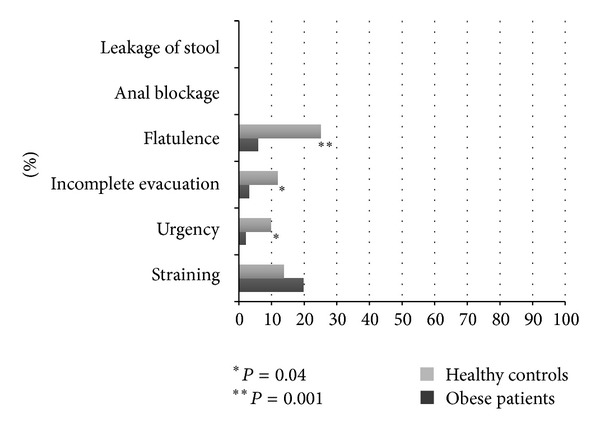
Prevalence of abdomino-pelvic symptoms such as leakage of stool, anal blockage, flatulence, incomplete evacuation, urgency, and straining in obese patients and HC. Data are expressed as percentages (%).

The prevalence of the other abdomino-pelvic symptoms such as leakage of stool, anal blockage, flatulence, incomplete evacuation, urgency, and straining in obese patients and HC is shown in Figure 3.

The prevalence of IBS diagnosis was 4/22 (18.2%) in obese patients with BE behavior pattern and 8/78 (10.3%) in obese patients without BE behavior pattern (*P* = 0.3). In detail, 3 obese patients with BE behavior pattern and 5 obese patients without BE behavior pattern fulfilled the diagnostic criteria for IBS-C; one obese patient with BE behavior pattern and 2 obese patients without BE behavior pattern for IBS-D and one obese patient with BE behavior pattern and no obese patients without BE behavior pattern for IBS-M (*P* = 0.75). None of the obese patients with BE behavior pattern were scored for Functional Constipation and 9/78 (11.5%) obese patients without BE behavior pattern were diagnosed with Functional Constipation (*P* = 0.2); 1/22 (4.5%) obese patients with BE behavior and no obese patients without BE behavior pattern fulfilled the diagnostic criteria for Functional Diarrhea (*P* = 0.5). Functional Bloating was found in none of the obese patients with BE behavior pattern and in 2/78 (2.6%) obese patients without BE behavior pattern (*P* = 0.9).

**Figure 4:**
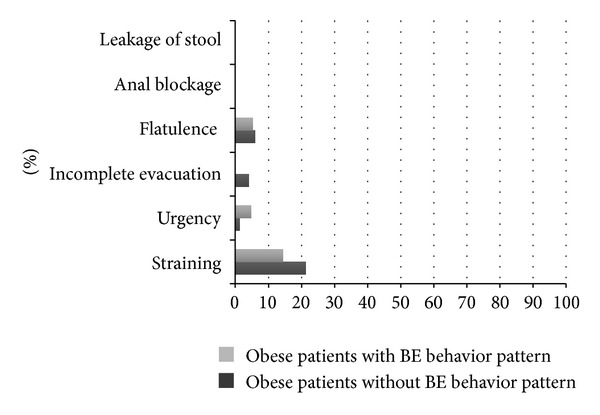
Prevalence of abdomino-pelvic symptoms such as leakage of stool, anal blockage, flatulence, incomplete evacuation, urgency, and straining in obese patients with and without BE behavior pattern. Data are expressed as percentages (%).

Figure 4 shows the prevalence of abdomino-pelvic symptoms in obese patients classified with and without BE behavior pattern.

## 4. Discussion

To our knowledge, this is the first study that evaluates a morbidly obese population from the south of Italy and the prevalence of FGID according to the internationally accepted Rome III criteria taking also into account the influence of BE behavior pattern. Our novel findings are a significantly higher prevalence of postprandial distress syndrome in obese patients with BE behavior pattern compared to obese patients without BE behavior pattern, whilst the prevalence of FGID in obese patients did not differ from controls. 

In detail, we found that the frequency of FD properly diagnosed according to Rome III criteria is the same in obese patients from the south of Italy as in control group. All obese patients of our series underwent EGDS, then FD was diagnosed after investigation excluding identified causal abnormalities. A similar frequency of FD according to Rome III has been recently reported by another Italian group in morbid obese patients prior to undergoing sleeve gastrectomy [26]. In contrast with our results, a previous study reported in obese patients a high prevalence of functional upper and lower disorders [13]. This contrast may be explained by the fact that the population of that study appeared to be less homogeneous in race, less than 10% of the patients were males, and there was no comparison with a control group. In our study, among the four cardinal dyspeptic symptoms identified by the Rome III committee, the frequency-intensity score of epigastric burning was significantly higher in obese population than controls, while early satiation was significantly lower. In addition, typical GERD symptoms, bloating, and abdominal pressure frequency-intensity scores appeared significantly increased in obese population in comparison to controls, and a significant correlation was found between increased upper GI symptoms and heartburn and regurgitation. This increased frequency of typical GERD symptoms is not surprising given the demonstrated association between GERD and obesity; however, it could explain the expansion of the whole spectrum of GERD-related symptoms. In fact, a significant increase in the upper abdominal sensations such as epigastric pressure, epigastric fullness, and nausea has previously been demonstrated in obese patients with abnormal esophageal exposure to acid, suggesting that the increased esophageal acid exposure in these patients might potentiate the perception of a different concurrent stimulus, such as barostat distensions [27]. As the Rome III committee did not consider heartburn and regurgitation symptoms that primarily arise from the gastroduodenum, even though they may occur simultaneously with gastroduodenal symptoms, our findings are still consistent with a prevalence of FD in obese patients similar to controls. 

From our data, it is noteworthy that early satiation is not a feature of obese patients of our series. This finding is consistent with the datum that satiation signals that inhibit ingestion are reduced with increased body mass index [28]. 

Our findings show a similar prevalence of IBS, Functional Constipation, Functional Diarrhea, and Functional Bloating in our obese population compared to controls. In the past few years other studies have found conflicting results about the prevalence of IBS in morbidly obese patients compared to the general population [11, 13, 29]. In population-based studies, obesity and being overweight have also been linked to other symptoms such as diarrhea [12]; conversely, no significant association between constipation/hard stools or bloating and increasing BMI was found [14]. Therefore, the association between obesity and functional bowel disorders has not yet been elucidated. Possibly, the differences in geographical origin and the socioeconomic and cultural conditions (quality and quantity of certain foods, FODMAPs etc.) might further contribute to explaining these discordances. Another strength of our study is that epidemiological studies in Italy demonstrated a prevalence of IBS similar to that found in our obese population [30].

We also evaluated in our study the prevalence of anorectal symptoms. No one reported fecal incontinence, in contrast with other findings that reported about 25% of it in morbidly obese patients before bariatric surgery [31]. On the other hand, a recent meta-analysis showed that the association between fecal incontinence and obesity was not statistically significant [14]. In addition, we found that none of our obese patients referred anal blockage, as previously reported [14]. The significant decrease in the frequency-intensity score of flatulence in our obese population also unlikely makes a high frequency of functional anorectal disorders in our obese population as recently suggested (article in press). However, the absence of the diagnostic tests needed to diagnose functional defecation disorders according to Rome III Criteria, might have been considered a limitation of the study.

Furthermore, in an attempt to investigate the possible role of BE behavior in the development of upper and lower GI symptoms, we further studied the association between the prevalence of the BE behavior pattern and FGID. A significant association between obese patients with BE behavior and PDS was demonstrated, while no obese patients with BE behavior fulfilled the diagnostic criteria for EPS. Moreover, obese patients with a BE behavior pattern showed a significant higher frequency-intensity scores for epigastric fullness and nausea compared to obese patients without BE behavior; whereas, the prevalence of GERD symptoms and their frequency-intensity scores were not significantly different between patients with and without BE behavior pattern. 

Also, no differences were found in the prevalence of IBS, Functional Constipation, Functional Diarrhea, and Functional Bloating, in patients with and without a BE behavior pattern. It has been previously demonstrated that BE disorder (BED) occurs in a subset ranging from 27% to 47% in severely obese persons undergoing bariatric surgery [15]. The effect of specific patterns of eating behavior such as BE on the development of both upper and lower GI symptoms in obesity has yet to be completely defined [16]. Features of BED include eating a large amount of food in a short period of time, eating rapidly, and experiencing a perceived loss of control over eating. From a physiological perspective, excessive intake of food over a relatively short time could potentially overcome the functional accommodation and emptying, contributing to the genesis of GI symptoms in obese individuals [16]. In our study, the presence of symptoms such as epigastric fullness and the lack of symptoms fulfilling the epigastric pain syndrome in our obese patients with a BE behavior pattern might suggest that the features of meal-related dyspeptic symptoms could play a role.

### 4.1. Study Limitations

Although the current study provides important insights regarding the prevalence of FGID in morbid obese patients and the potential impact of BE behavior on these diagnoses, there are a number of limitations. First, the population surveyed in the present sample was Italian, specifically from the south of Italy, and afferent to a tertiary centre devoted to bariatric surgery. Thus, the results might not generalize to other ancestry groups and to the general population. Second, this study was a case-control study. It is therefore important to recognize that here we elucidated a significant association, but a causal relationship among them cannot be drawn. This limitation also highlights the importance of research designs in determining the pathophysiological mechanisms underlying the associations between BE behavior, obesity, and the onset of GI symptoms. Another potential confounder could be other diseases that might be associated with both obesity and GI symptoms. Diabetes mellitus is strongly associated with obesity, but its relationship with GI symptoms remains unclear. In this study the prevalence of diabetes was low (4%) and the results did not change when excluding these subjects from the analysis (data not shown).

In conclusion, the results of the current study suggest that clinical practice might benefit from a more regular screening of PDS when evaluating obese patients presenting with GI symptoms to disclose BED. The management of any relevant BED should be incorporated into designing and tailoring future treatment interventions, given that the continued presence of this behavior may contribute to upper GI symptoms and, consequently, prevent optimum treatment outcomes. 

Further research is urgently required on this important topic in obese patients that may assist in understanding the pathophysiological interactions between obesity and GI symptoms. 

## Figures and Tables

**Table 1:** Demographic characteristics, anthropometric data, and prevalence of comorbidities and of BE behavior pattern in the Italian obese patients and healthy controls. Data are expressed as percentage (%) or as mean ± SE.

	Obese patients *n* = 100	Healthy controls *n* = 100	*P*
Gender (M/F)	44/56	36/64	0.3
Age (years)	34.3 ± 1.1	31.2 ± 1.6	0.1
Weight (Kg)	131.3 ± 2.9	64.9 ± 1.8	<0.001
BMI (Kg/m^2^)	45.9 ± 0.7	23.4 ± 0.4	<0.001
Ethnic origin (Caucasian %)	100	100	
Smoking (%)	31.0%	14.0%	0.007
Number of cigarettes per day	23.9 ± 2.7	7.4 ± 3.1	0.004
Diabetes (%)	4	0	
Hypertension (%)	19.0	0	
Dyslipidemia (%)	17	0	
Respiratory diseases (%)	41	0	
Musculoskeletal disorders (%)	21	0	
BE behavior pattern (%)	22.0	0	<0.001

M: male; F: female; BMI: body mass index.

**Table 2:** Shows the frequency intensity scores of the studied GI symptoms such as epigastric pressure, belching, nausea, cough, non cardiac chest pain, dysphagia for liquids, dysphagia for solids, regurgitation, and heartburn in Obese Patients with and without BE behavior pattern data were expressed as Mean ± SE.

	BE behavior pattern	No BE behavior pattern	*P*
Epigastric pressure	0.57 ± 0.20	0.40 ± 0.14	0.56
Belching	0.38 ± 0.18	0.53 ± 0.15	0.62
Nausea	0.19 ± 0.13	0.03 ± 0.03	0.05
Cough	0.09 ± 0.09	0.12 ± 0.09	0.88
Non cardiac chest pain	0.18 ± 0.12	0.08 ± 0.06	0.41
Dysphagia for liquids	0.00 ± 0.00	0.03 ± 0.02	0.60
Dysphagia for solids	0.00 ± 0.00	0.13 ± 0.08	0.37
Regurgitation	0.91 ± 0.36	1.27 ± 0.22	0.43
Heartburn	1.00 ± 0.37	1.26 ± 0.20	0.55
